# The ups and downs of intermittent hypoxia as a therapy for ventilatory insufficiency

**DOI:** 10.1113/JP283143

**Published:** 2022-04-28

**Authors:** Ken D. O'Halloran

**Affiliations:** ^1^ Department of Physiology, School of Medicine, College of Medicine & Health University College Cork Cork Ireland

**Keywords:** control of breathing, plasticity, therapeutic hypoxia

The respiratory control network is an exemplar of homeostatic regulation. Notwithstanding the principal function of orchestrating appropriate adjustments in alveolar ventilation to maintain tight regulation of blood gas tensions and pH, a wealth of experimental evidence convincingly demonstrates the considerable capacity for plasticity at multiple sites of the network, in a variety of settings in health and disease. Such changes are at times an integral component of the physiological response to stressors that includes, for example, environmental stress such as exposure to high altitude. It follows that aberrant plasticity can also manifest in some scenarios, resulting in overt cardiorespiratory malaise.

Since the seminal observation by Millhorn et al. (1980) of lasting potentiation of respiratory motor outflow following repeated carotid sinus nerve stimulation, a rich legacy of research has carefully documented the phenomenon of long‐term facilitation of breathing, in its many guises. Various paradigms of acute exposure to repeated bouts of hypoxia have been shown, in several species, to evoke persistent elevation in respiratory motor outflow(s) and/or breathing, lasting at least one hour following termination of the antecedent recurrent stimulus. Whereas early reports revealed potentiation of respiratory motor outflow at the level of the respective motor nuclei, it was subsequently demonstrated that facilitated breathing can manifest owing to plasticity at multiple sites of the reflex chemosensory pathway. And whereas intermittent hypoxia has most commonly been studied, and is often sufficient to elicit long‐term facilitation, there is a growing recognition of the potential benefit, and apparent necessity in humans, of concurrent isocapnia or hypercapnia, consistent with the pivotal role of CO_2_ in the regulation of respiration. Fundamental research of experimental protocols evoking forms of respiratory plasticity in animal models has translated to potential clinical application, with explorations of the efficacy of therapeutic intermittent hypoxia in pre‐clinical animal models and human trials, including promising studies in people with spinal cord injury (Christiansen et al., 2021) and amyotrophic lateral sclerosis (Sajjadi et al., 2022). There remains a need to fully characterise the capacity for evoked plasticity in the respiratory control network and to further explore effective experimental protocols of potential translational value.

In this issue of *The Journal of Physiology*, Welch et al. (2022) compared the efficacy of an intermittent hypoxia protocol with and without attendant hypercapnia to elicit lasting facilitation of corticospinal neurotransmission in healthy awake human participants. Using surface electromyography, the authors demonstrated increased diaphragmatic motor‐evoked potentials induced by transcranial magnetic stimulation, but interestingly, no change in compound muscle action potentials induced by cervical magnetic stimulation. Potentiated motor‐evoked potentials were observed 60 min following hypercapnic hypoxia, but not poikilocapnic hypoxia trials, which were equivalent to sham control trials. Mouth occlusion pressure at 0.1 s, a validated index of respiratory drive, was also elevated following intermittent hypercapnic hypoxia exposure, but neither intermittent hypercapnic hypoxia nor intermittent poikilocapnic hypoxia elicited long‐term facilitation of ventilation (normalised to metabolic CO_2_ production).

Thus, the scope for intermittent hypoxia‐dependent plasticity extends to corticospinal circuitry but appears dependent upon concurrent exposure to CO_2_ during repeated bouts of hypoxia. Stimulus interaction between hypoxia and hypercapnia at the peripheral chemoreceptors of the carotid body is well recognised, resulting in hyper‐additive sensory signalling to the brain and greatly potentiated cardiorespiratory outputs. Multiple brain regions show intrinsic CO_2_ chemosensitivity and a capacity to shape the rhythm and pattern of breathing during hypercapnia. The mechanism driving corticospinal facilitation following exposure to hypercapnic hypoxia remains unclear but requires attention so that it can be understood and potentially better harnessed.

Both protocols failed to elicit long‐term facilitation of ventilation. Whereas others too have shown in human participants that intermittent hypoxia alone may be insufficient to drive persistent facilitation of breathing, Vermeulen et al. (2020) recently reported long‐term facilitation of breathing following intermittent hypercapnic hypoxia, employing a protocol reminiscent of human sleep apnoea, wherein the frequency of exposure to repeated bouts is higher than that typically employed in studies focused on therapeutic application. In obstructive sleep apnoea, repeated occlusions of the upper airway during sleep perturb airflow resulting in combined hypoxia and hypercapnia. Whether long‐term facilitation of breathing in the context of sleep apnoea is beneficial or harmful is arguable. Whereas it may enhance respiratory motor drive to upper airway muscles stabilizing upper airway calibre, it can also serve to destabilize breathing and potentially perpetuate recurrent apnoea. Moreover, symapthoexcitation provides a substrate for neurogenic hypertension.

In the context of ventilatory insufficiency, which is common in people with cervical spinal cord injury and various neuromuscular disorders, interventions that facilitate respiratory motor outflow may counter respiratory morbidity and improve quality of life. As such, delineation and refinement of optimal protocols of intermittent hypercapnic hypoxia are clearly warranted but may have some inherent limitations. Notably, Welch et al. (2022) highlight that self‐reported discomfort scores were elevated when participants breathed hypercapnic hypoxia, which could provoke dyspnoea in some people with respiratory dysfunction. Whereas this too warrants consideration in the context of interventional therapy, cumulative exposure time during therapeutic sessions is short. Moreover, it may be that isocapnic hypoxia is adequate to evoke ventilatory long‐term facilitation (Keough et al., 2021), depending on the frequency, intensity and duration of the stimulus. Additional considerations arise in the context of recurrent therapeutic sessions, relevant to both respiratory sensation and the expression of long‐term facilitation, all of which must be established if the concerted ambition to develop an effective therapy is to be realised.

Whilst boosting motor signals in spared pathways in spinal cord injury or motor neuron disease is likely to prove advantageous, the potential trade‐off between benefit and injury of facilitated motor drive in respiratory pathways in some neuromuscular diseases, such as muscular dystrophy, requires careful consideration. Muscular dystrophies are characterised by respiratory insufficiency. However, increased mechanical work by respiratory muscles may aggravate muscle pathology, for example due to contraction‐induced injury, particularly relevant to Duchenne muscular dystrophy. In addition to the continued careful attention to detail required to avoid pathophysiological consequences of intermittent (hypercapnic) hypoxia, it will also be important to determine the breadth and boundaries of clinical application. The evolving narrative portrays a wonderful example of the translation of a curious observation in the laboratory to a potential therapeutic modality. Sapere vedere!

## Additional information

### Competing interests

None.

### Author contributions

Sole author.

### Funding

Science Foundation Ireland (SFI): Ken D. O'Halloran, SFI FFP 2019/6628.

## Supporting information


Peer Review History
