# Supplementary material for: The ups and downs of intermittent hypoxia as a therapy for ventilatory insufficiency
Source: J Physiol. 2022 Apr 28;600(10):2275–6. doi: 10.1113/JP283143 (PMC9325070; doi:10.1113/JP283143)
Supplement: Supplementary file 1 — Peer Review History [file TJP-600-2275-s001.pdf]

The Journal of Physiology

The ups and downs of intermittent hypoxia as a therapy for ventilatory insufficiency

Ken D O'Halloran
DOI: 10.1113/JP283143

Corresponding author(s): Ken O'Halloran (k.ohalloran@ucc.ie)

The referees have opted to remain anonymous.

Review Timeline:

Submission Date:	08-Apr-2022
Accepted:	11-Apr-2022

Senior Editor: Scott Powers

Reviewing Editor: Scott Powers

Transaction Report:

(Note: With the exception of the correction of typographical or spelling errors that could be a source of ambiguity, letters and reports are not edited. Depending on transfer agreements, referee reports obtained elsewhere may or may not be included in this compilation. Referee reports are anonymous unless the Referee chooses to sign their reports.)

1st Editorial Decision 11-Apr-2022

Dear Professor O'Halloran,

Re: JP-TP-2022-283143 "The ups and downs of intermittent hypoxia as a therapy for ventilatory insufficiency" by Ken D O'Halloran

I am pleased to tell you that your invited Translational Perspective has been accepted for publication in The Journal of Physiology, subject to any modifications that may be required by the Journal Office to conform to House rules.

NEW POLICY: In order to improve the transparency of its peer review process The Journal of Physiology publishes online as supporting information the peer review history of all articles accepted for publication. Readers will have access to decision letters, including all Editors' comments and referee reports, for each version of the manuscript and any author responses to peer review comments. Referees can decide whether or not they wish to be named on the peer review history document.

The last Word version of the paper submitted will be used by the Production Editors to prepare your proof. When this is ready you will receive an email containing a link to Wiley's Online Proofing System. The proof should be checked and corrected as quickly as possible.

All queries at proof stage should be sent to tjp@wiley.com

Thank you for your contribution to The Journal of Physiology.

Yours sincerely,

Scott K. Powers
Senior Editor
The Journal of Physiology
https://jp.msubmit.net
http://jp.physoc.org
The Physiological Society
Hodgkin Huxley House
30 Farringdon Lane
London, EC1R 3AW
UK
http://www.physoc.org
http://journals.physoc.org

****

Reviewing Editor Comments:

Thank you for submitting this excellent TP to the Journal of Physiology. This is a thoughtful and and well-written report. Congratulations on this outstanding contribution to JP.

Senior Editor Comments:

Thank you for this outstanding contribution to the Journal of Physiology.

Confidential Review 08-Apr-2022
